# Anxiety, insomnia and family support in nurses, two years after the onset of the pandemic crisis

**DOI:** 10.3934/publichealth.2023019

**Published:** 2023-04-21

**Authors:** Christos Sikaras, Maria Tsironi, Sofia Zyga, Aspasia Panagiotou

**Affiliations:** 1 University of the Peloponnese, Department of Nursing, 22100, Tripoli, Greece; 2 Sotiria Thoracic Diseases Hospital of Athens, Nursing Department, 11527, Athens, Greece

**Keywords:** anxiety, insomnia, family support, nurses, COVID-19, mediation analysis

## Abstract

**Introduction:**

The Covid-19 pandemic continues to cause serious physical and mental problems for health professionals, particularly nurses.

**Aim:**

To estimate the prevalence of anxiety and insomnia and to evaluate their possible association with family support received by nurses two years after the onset of the pandemic.

**Materials and methods:**

In total, the study participants were 404 nurses (335 females and 69 males) with a mean age of 42.88 (SD = 10.9) years and a mean of 17.96 (SD = 12) years working as nurses. Nurses from five tertiary hospitals in Athens constituted the study population who completed the questionnaires State-Trait Anxiety Inventory (STAI), Athens Insomnia Scale (AIS) and Family Support Scale (FSS), in the months of November and December 2021. Regarding demographic and occupational characteristics, gender, age and years of experience as nurses were recorded.

**Results:**

60.1% of the nurses showed abnormal scores in state anxiety, with 46.8% in trait anxiety, and 61.4% showed insomnia. Women showed higher scores on the two subscales of anxiety and the insomnia scale compared to men (p < 0.01 and p < 0.05 respectively), while they showed a lower score on the FSS without statistical significance (p > 0.05). Positive correlations (p < 0.01) were found between the State Anxiety Inventory, Trait Anxiety Inventory and AIS, while all of them showed a high negative correlation with FSS (p < 0.01). Age showed a negative correlation with Trait Anxiety Inventory (p < 0.05). As shown by the mediation analysis, the relationship between state anxiety and insomnia was mediated by trait anxiety, whereas state anxiety appeared to be dependent on family support.

**Conclusions:**

Nurses continue to experience high levels of anxiety and insomnia and feel less supported by their families than in the first year of the pandemic. Insomnia appears to be dependent on state anxiety, with a significant indirect effect of trait anxiety, while family support seems to affect state anxiety.

## 1. Introduction

Since the beginning of the 21st century, the emergence of infectious disease epidemics has become increasingly frequent and complex, endangering the health of the general population and causing psychological problems [1], especially among healthcare workers [1].

The COVID-19 pandemic is the culmination of these, causing various physical and psychological problems in different populations [2],[3] and especially in health professionals [2]–[4], with the frequency and severity of physical and psychological problems in them being higher than in previous epidemics [5].

According to recent studies, nurses had a higher rate of emotional symptoms than other healthcare workers [6]–[8]. Nurses represent the largest proportion of frontline health professionals who provide services to patients [9] and are a special professional group that plays a very important role in the proper functioning of health care systems, performing a particularly demanding profession that requires high physical and mental efforts [9]. Due to various factors that cause stress, such as frequent shift work, increased workload and/or contact with death, nurses experience intense anxiety, resulting in both a possible decline in their quality of life and a reduction in of the quality of care they provide to patients [10]. According to cognitive theory, regarding the relationship between trait anxiety and state anxiety, stressful events in individuals activate some stable underlying maladaptive dysfunctional self-schemas. According to the evidence, stressful events usually precede insomnia. However, it is possible that there are some predisposing factors that are responsible for the development of insomnia, because not everyone who experiences stressful events develops insomnia [11].

According to literature data, poor sleep quality is related to anxiety [12], which is the main cause of sleep disorders in healthcare workers [13],[14]. In addition, in nurses, due to their frequent work in rotating shifts, sleep disorders have a high prevalence, with morbid consequences for their health [15],[16]. Their increased work responsibilities during the pandemic crisis caused an aggravation of the already problematic and stressful working conditions [8], resulting in the possible worsening of sleep disorders in the long term due to the additional stress [17]. We should also take seriously the fact that sleep is necessary to maintain life, and according to recent literature data, in order to achieve optimal health in adults, 7 to 9 hours of sleep are required [18].

Another important protective factor for the mental health of healthcare professionals is perceived social support. Its lack, in the conditions of the pandemic, is exacerbated by social distancing, social stigmatization and the fear of contagion of familiar persons, and this lack has a negative impact on the mental health of health professionals [6]. According to recent studies during the pandemic, perceived social support appeared to have a strong protective effect on the mental health of healthcare professionals and was found to be associated with lower anxiety and insomnia [4],[19].

One of the most important components of social support is the family [20]. Family support is the support a person perceives they receive from their family. Its role seems to be particularly important in situations that require social distancing [21]. Compared to other societies, it seems to play a more important role in the Greek population due to particularly close family relationships [22].

## 2. Aim

While the COVID-19 pandemic is still ongoing, the aim of our study was to estimate the prevalence of anxiety and insomnia and to evaluate their possible association with family support received by nurses two years after its onset.

Further research questions were posed:

1. What are the levels of anxiety, insomnia and family support received by nurses?

2. Are anxiety, insomnia and family support related to each other?

3. Does family support affect the relationship between anxiety and insomnia?

## 3. Material and methods

### 3.1. Study design and setting

This was a cross-sectional observational and correlational study. Data were collected through questionnaires which were distributed by the researchers to the study participants. One hundred questionnaires were distributed to each hospital participating in the study. To further ensure the confidentiality and anonymity of the participants, the questionnaires were distributed by the researcher to the nursing staff of the nursing departments in an envelope with a self-adhesive closure, at their place of work. A box was placed in each nursing department to collect the envelopes with the completed questionnaires. All the nursing staff of each department had free access and the possibility to complete the questionnaire. Participants were informed about the purpose of the study. Questionnaires were anonymous, and participants had the right to voluntarily withdraw from the study at any time. The participation of nurses in the study was voluntary. As a criterion for inclusion in the study group, the existence of professional experience of at least one year was defined.

### 3.2. Participants

The required sample size, taking into account the target population [11], with a 95% confidence level, a 5% margin of error and a percentage of 50%, was set at a minimum of 379 participants [23]. From five tertiary hospitals in Athens, a total of 404 questionnaires were filled out by the nurses out of the 500 that had been distributed (80.80% response rate). The participants worked in departments providing care to patients with COVID-19 but also in departments that treated patients with other diseases, such as surgical and pathology departments, intensive care units (ICU) or in other nursing departments. Regarding demographic and occupational characteristics, gender, age and years of experience as nurses were recorded. The survey was conducted in the months of November and December 2021.

### 3.3. Measurement tools

#### 3.3.1. The Spielberger State-Trait Anxiety Inventory (STAI-Y Form)

This scale was used to assess anxiety. It is one of the most commonly used scales and distinguishes anxiety into state anxiety and trait anxiety. It consists of two subscales: the subscale that assesses anxiety caused by a specific situation (State Anxiety) and the subscale that assesses anxiety that is a more permanent characteristic (Trait Anxiety). Each subscale consists of 20 items, and each item is scored from 1 to 4, with the total score for each subscale ranging from 20 to 80. Higher scores indicate higher levels of anxiety [24].

The Greek version of the scale, which was used in our study, is a reliable tool for assessing anxiety. In the State Anxiety subscale (STAI Form Y-1), subjects assess how the respondent feels at the moment. The answers include the answers “not at all, a little, moderately, a lot” and the respondent chooses the answer that best describes his feelings. In the Trait Anxiety subscale (STAI Form Y-2), the items assess how the respondent feels in general. The answers include the answers “not at all, a little, moderately, a lot” and the respondent chooses the answer that best describes his feelings. In both subscales, each item is scored from 1 to 4. For the State Anxiety subscale the internal consistency (Cronbach's alpha) was 0.93, while for the Trait Anxiety subscale it was 0.92 [25].

#### 3.3.2. Athens Insomnia Scale (AIS)

The Athens Insomnia Scale assesses difficulty in sleeping. The scale was constructed by Greek researchers in the Greek language and has been used in many studies of health professionals [7],[21]. It consists of eight items that assess the quality and duration of sleep during the night, as well as how the respondent feels during the following day. The first five items concern sleep induction, awakenings during the night, final awakening, total sleep duration and sleep quality, while the last three items are related to dysfunction during the next day. The score for each topic ranges from 0 (no problem) to 3 (severe problem). The total score ranges from 0 to 24. Sleep is finally evaluated by the cumulative score of all items. A score ≥ 6 is used for the clinical diagnosis of insomnia [26]. For the total scale, the internal consistency (Cronbach's alpha) was 0.89 [27].

#### 3.3.3. Family Support Scale (FSS)

This scale assesses family support, i.e., the individual's feeling of being supported by their family. It consists of 13 questions scored from 1 to 5 and a question indicating the number of people living in the household of the person completing the questionnaire [28]. In the present study, this scale was used in the Greek version. This scale has been used in many studies, both in patients [22],[29] and nurses [21]. It is a reliable and brief tool for assessing family support. Generally, higher scores indicate greater levels of family support [30].

### 3.4. Ethics approval of research

This study was conducted in accordance with the ethical principles as defined by the Declaration of Helsinki, the International Committee of Medical Journal Editors and the General Data Protection Regulation (GDPR-2016/679) of the European Union. The study protocol was approved by the Ethics Committee of the Peloponnese University (18699/11–10–2021), as well as by the Clinical Research Committees of the hospitals that were included in this study.

### 3.5. Statistical analysis

We used descriptive statistics to evaluate all variables. Mean and standard deviation were calculated for continuous variables. We determined the prevalence of both anxiety and insomnia as percentages. We used sample and independent t-tests to examine mean differences between groups. To examine correlations between continuous variables, the Pearson test was performed. We constructed a linear regression model to investigate whether the correlated variables explained the variance of the Insomnia Scale, and we evaluated the linear regression assumptions before creating the model. We used macros 4 and 5 of the Hayes SPSS procedure to perform mediation and moderation analyses. Analyses were performed using SPSS-23, and statistical significance was determined at p < 0.05 (two-tailed).

## 4. Results

In total, the study participants were 404 nurses (335 females and 69 males) aged 22 to 62 years, with a mean of 42.88 (SD = 10.9) years, and 1 to 40 years working as nurses, with a mean of 17.96 (SD = 12) years (Table 1).

**Table 1.** General characteristics of nurses and Anxiety/Insomnia/Family Support scores in relation to gender.

Participants	Descriptive statistics	Age	Work experience (in years)	State Anxiety Inventory	Trait Anxiety Inventory	Athens Insomnia Scale	Family Support Scale
Male N = 69	Mean	41.16	15.60	35.46**	36.32**	6.12*	50.70
	SD	11.37	11.67	12.14	11.35	4.64	7.62
Female N = 335	Mean	43.23	18.45	40.18**	40.30**	7.36*	48.66
	SD	10.79	12.02	11.59	10.52	4.25	8.49
Total N = 404	Mean	42.88	17.96	39.37	39.62	7.15	49.00
	SD	10.90	12.00	11.80	10.76	4.34	8.29

Notes: *p < 0.05; **p < 0.01.

The descriptive statistics of the scales in relation to gender are presented in Table 1. The assessment of the results showed that women had higher mean scores on the two anxiety subscales and the insomnia scale compared to men with statistical significance, while they showed a lower mean score on the family support scale with no statistical significance (Table 1).

Regarding anxiety, 60.1% of the nurses had a pathological score on state anxiety, and 46.8% had such a score on trait anxiety. Also, 61.4% of nurses experienced insomnia.

The mean of insomnia (AIS: Mean = 7.15) in our study was statistically greater (sample t-test, p < 0.01) than the mean of insomnia (AIS: Mean = 5.98, SD = 4.24, N = 150) experienced by Greek hospital nurses during the first pandemic wave [21]. Calculating Hedges' g between the previous measurement and the present, a small effect size was found (g: 0.269).

Regarding the scale of family support, the mean score (FSS: Mean = 49.00) in our study was statistically lower (sample t-test, p < 0.01), compared to the mean (FSS: Mean = 52.67, SD = 7.99, N = 150) presented by the nurses of Greek hospitals during the first pandemic wave [21]. Calculating Hedges' g between the previous measurement and the present, a medium effect size was found (g: 0.447).

Between Trait Anxiety Inventory, State Anxiety Inventory and Athens Insomnia Scale, positive correlations were found (p < 0.01, Table 2), while all three scales showed a high negative correlation with the Family Support Scale (p < 0.01, Table 2). Age was also found to show a negative correlation with the Trait Anxiety Inventory (p < 0.01, Table 2).

**Table 2.** Correlations among age, work experience, State Anxiety Inventory, Trait Anxiety Inventory, Athens Insomnia Scale and Family Support Scale.

Pearson Correlation N = 404		Age	Work Experience (in years)	State Anxiety Inventory	Trait Anxiety Inventory	Athens Insomnia Scale
Work Experience (in Years)	r	0.885**				
	p	0.001				
State Anxiety Inventory	r	−0.056	−0.082			
	p	0.258	0.104			
Trait Anxiety Inventory	r	−0.101*	−0.060	0.789**		
	p	0.043	0.232	0.001		
Athens Insomnia Scale	r	−0.034	−0.094	0.544**	0.604**	
	p	0.496	0.064	0.001	0.001	
Family Support Scale	r	0.030	0.051	−0.431**	−0.502**	−0.355**
	p	0.596	0.366	0.001	0.001	0.001

Notes: *Correlation is significant at the p < 0.05 level (two-tailed); ** Correlation is significant at the p < 0.001 level (two-tailed).

In order to identify the best predictors of insomnia, stepwise multiple regression analysis was then performed. With insomnia as the dependent variable, sex, age, years of work experience, Trait Anxiety Inventory, State Anxiety Inventory and Family Support Scale were entered as independent variables. Stepwise multiple regression showed that 36.5% of the variance in the Athens Insomnia Scale score could be explained by Trait Anxiety Inventory, while an additional 2.2% could be explained by State Anxiety Inventory (Table 3). All other variables did not explain the variance in the Athens Insomnia Scale score.

**Table 3.** Stepwise multiple regression (only statistically significant variables are included).

Dependent Variable: Athens Insomnia Scale	R Square	R Square Change	Beta	t	p	Durbin-Watson
Spielberger Trait Anxiety Inventory	0.365	0.365	0.419	5.96	0.001*	2.147
Spielberger State Anxiety Inventory	0.387	0.022	0.238	3.38	0.001*	

Notes: Beta = standardized regression coefficient; *Correlations are statistically significant at the p < 0.001 level.

**Figure 1.**
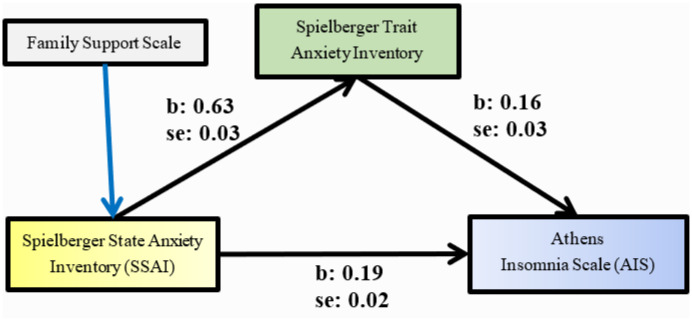
Mediation analysis of Spielberger Trait Anxiety Inventory on Spielberger State Anxiety Inventory – Athens Insomnia Scale (AIS) relationship.

In order to examine whether trait anxiety mediates the relationship between state anxiety and the Athens Insomnia Scale, we performed bootstrapping with the Hayes SPSS Process Macro. Based on 5000 bootstrap samples, we found that the statistically significant indirect relationship between State Anxiety Inventory and the Athens Insomnia Scale was mediated by Trait Anxiety Inventory (Table 4, Figure 1). In particular, the indirect effect of Trait Anxiety Inventory was found to be statistically significant [B = 0.1354, 95% CI (0.1035, 17.24), p < 0.05]. Gender, age, years of work experience and Family Support Scale were included as covariates. State Anxiety Inventory seems to depend on Family Support Scale (Table 4), while the other variables that were used as covariates did not show a statistically significant effect.

**Table 4.** Mediation analysis of Spielberger Tait Anxiety Inventory (STAI) on Spielberger State Anxiety Inventory (SSAI) – Athens Insomnia Scale (AIS) relationship and Family Support Scale (FSS) as covariate variable.

Variable	b	SE	t	p	95% Confidence Interval	
					LLCI	ULCI
SSAI →STAI	0.6312	0.0345	18.3018	0.001	0.5634	0.6991
SSAI → AIS	0.1872	0.0190	9.8596	0.001	0.1499	0.2246
SSAI → STAI → AIS	0.1621	0.0298	5.4428	0.001	0.1387	0.2412
FSS→ SSAI	−0.0840	0.0333	−2.5240	0.04	−0.1495	−0.0185
Effects
Direct	0.0849	0.0262	3.2458	0.005	0.0334	0.1364
Indirect**	0.1023	0.0178			0.0969	0.1390
Total	0.1872	0.0190	9.8596	0.001	0.1499	0.2246

Note: *Gender, age and years of work experience were included in the analysis as covariate variables. They are not shown in the table because they did not give statistically significant results (p > 0.05); **Based on 5000 bootstrap samples.

In the end, the mediating role of the Family Support Scale in the relationship between the State Anxiety Inventory and the Athens Insomnia Scale was evaluated. Retention analysis was performed using model 5 of the PROCESS method. The Family Support Scale showed a non-statistically significant moderating role in the relationship between State Anxiety Inventory and the Athens Insomnia Scale (p > 0.05).

## 5. Discussion

The COVID-19 pandemic, having started almost three years ago (early 2020), is now a chronic stressor that is still ongoing, with the long-term psychological impact on health professionals remaining unknown [31],[32]. Studies have reported that the psychological effects of past epidemics (including SARS) on health professionals lasted up to 3 years after their onset [33].

The results showed that women showed higher levels of both state and trait anxiety compared to their male counterparts, a finding consistent with numerous other studies [7],[9],[34]–[36]. According to the literature, sex hormones, particularly estradiol and progesterone, play an important role in women's vulnerability to anxiety [37].

Regarding insomnia, women had higher levels of insomnia compared to men, a finding that agrees with other recent studies [38]–[41]. Although it is not clear why women have a higher prevalence of sleep disorders than men, one possible explanation could be that it is due to underlying structural differences in the brain between men and women [40],[42].

However, according to a recent study by AlRasheed et al., insomnia symptoms were not found to be related to gender [43]. According to Carmel et al. [44] women are affected differently than men by stressors and use coping resources differently. These findings are in agreement with the findings of other studies showing that female nurses are more vulnerable to mental health problems compared to males [45],[46]. Also, we should take seriously the fact that female nurses constitute the vast majority of the nursing staff, and therefore immediate measures should be taken for their psychosocial support [47].

Also, according to the results of our study, nursing staff showed very high levels of state anxiety (60.1%), trait anxiety (46.8%) and insomnia (61.4%). These rates are among the highest found compared to recent studies in other countries. In a recent systematic review [34] the prevalence of anxiety in nurses was found to be 31.93 % (27.44–36.42), and that of insomnia was 39.06 % (35.04–43.08). Similar results to our study were shown by the study of Jahrami et al. [48]. In the study of Pappa et al. [7] the rate of anxiety in nurses was found to be 25.80% (19.20–33.00), while insomnia overall in health care professionals was found to be 38.9%. In the umbrella review of meta-analyses by Sahebi et al. [48] insomnia overall in health professionals was found to be 36.36% (33,36–39).

Another important finding is that the mean of insomnia in the present study (AIS: 7.15) was higher with a statistically significant difference (sample t-test, p < 0.01) than the mean of insomnia (AIS: Mean = 5.98, SD = 4.24, N = 150) recorded in Greek hospitals during the first wave of the pandemic [21].

Similar results were shown in the study by Jahrami et al., who argued that sleep quality deteriorated two years after the onset of the pandemic crisis [49]. Also, a comparative study in Greece in the general population showed that sleep duration decreased in the second lockdown compared to the first lockdown, and sleep quality gradually deteriorated as the pandemic restrictions continued [50].

Moreover, it should be mentioned that during this study (November and December 2021), the Greek population was experiencing the peak of the fourth wave of the pandemic which caused a high mortality rate, with the inability of the national health system to respond fully [51], combined with a severe shortage of nurses [52].

Also, in the present study, associations between state anxiety, trait anxiety and insomnia were found to be strong, a finding that is consistent with numerous studies that support that anxiety and sleep disorders are related [12] and that anxiety is a major cause of disorders sleep in health professionals [13],[14]. Some recent studies suggest that anxiety and perceived stress have increased the incidence of sleep disorders in physicians and nursing staff during the pandemic crisis [53]–[57].

Stress and sleep are two factors that can have negative effects on each other [56]. The presence of insomnia can exacerbate coexisting psychiatric conditions, impede response to treatment and lead to an increased risk of recurrence of psychiatric comorbidities such as anxiety and depression [58]. Hertenstein et al. in a recent meta-analysis argued that insomnia is a significant predictor of anxiety [59], while a study by Meaklim et al. argued that insomnia constitutes a modifiable risk factor for anxiety and depression [60].

Our study also showed higher levels of state anxiety in younger nurses, a finding consistent with recent studies [61]–[63]. Considering that younger individuals have lower resilience [63],[64], age-sensitive interventions should be urgently developed to improve the modifiable factors contributing to their psychological distress [63],[64].

Another important finding of our study is the high negative association of family support with state anxiety, trait anxiety and insomnia. This finding of our study is consistent with the results of other studies which highlight the protective role of family support against negative psychological conditions both in nursing staff [65],[66] and in patients with chronic diseases [22],[67].

Recent studies during the pandemic crisis support the view that social support, of which family support is a key component, particularly in situations requiring social distancing such as the present one [21], serves as an important protective factor of mental health [68],[69].

Another important issue examined in this study is the decrease in perceived family support experienced by nurses in the second year of the pandemic crisis compared to the first year. To interpret this finding, we need to consider the social status experienced by nurses at the beginning of the pandemic where they were suddenly labeled as heroes by the general public, the media and politicians [70],[71]. Regardless of the reasons for this sudden heroization [70],[71], the event likely caused an increase in the family support provided to nurses [70]. As the second year of the pandemic dimmed, if not extinguished, the heroic element, family support returned to previous pre-pandemic lows [28]. This finding of our study is discouraging for the mental health of nurses, especially considering that family support—such as positive thinking, adequate sleep and spirituality—was one of the main coping strategies used by healthcare professionals during the pandemic [72],[73].

In our study, we did not consider other aspects that may influence nurses' anxiety and insomnia, such as marital status, workload, work department and education level. These factors were not included in this study and are possible confounding factors.

A study by Htay et al. during the pandemic showed that those who lived alone and those who worked in intensive care units had more severe symptoms of anxiety [74]. Also regarding workload, a study by Marzo et al. showed that health care workers who worked more than 10 hours per day had lower mean physical and psychological health scores than those who worked between 7 and 10 hours [75].

Regarding the level of education, although the results of studies are conflicting, the study by Marzo et al. showed that participants who had secondary education had higher physical and psychological health scores compared to those with tertiary education [75]. According to Marzo et al. this finding is probably due to the additional anxiety that those with tertiary education may experience due to the responsibility they have [75].

Finally, to address the psychological impact of the pandemic on nurses, mental health support should be encouraged and provided to all, especially those who need it most [72]–[74],[76].

Our study was based only on quantitative data and self-report scales. Given the multifaceted nature of the research problem, longitudinal and qualitative studies should be conducted in the future to provide additional information about nurses' experiences during the pandemic and to determine the relationships between risk factors and outcomes.

## 6. Strengths and limitations

Our study has some strong points, such as the following:

-According to the results of our study, nurses perceived that they received less family support in the second year of the pandemic compared to the first year. From the literature search, no other relevant study was found.

-Another important finding of our study is the worsening of insomnia two years after the onset of the pandemic, a finding that few studies have found.

These findings can be used as reference data to develop an effective support system to reduce anxiety, improve family support and improve nurses' sleep health.

Despite the strong theoretical and methodological background, the study had some limitations, including the following:

-While the study provides valuable information about nurses' experiences during the pandemic crisis, the results cannot be generalized to nurses throughout the country due to the limited sample size and the fact that the study was conducted in five tertiary public hospitals in Athens.

This limitation reduces the external validity of the study.

-Future longitudinal and qualitative studies could provide additional information about nurses' experiences during the pandemic crisis and determine the relationships between risk factors and outcomes.

-The specific factors related to each ward in which the nurses worked, due to their frequent rotations in different wards of the hospital as needed, were not included in this study. It should also be taken into account that at the time of the present study, Greece was experiencing the peak of the pandemic, with high mortality and inability of the national health system to respond fully [51], combined with a severe shortage of nurses [52], which had a negative impact on all nurses, regardless of the ward in which they worked. These limitations affected the internal validity of the study because they did not allow us to determine to what extent the high levels of anxiety and insomnia observed in this study (despite adequate personal protective equipment, unlike the first wave of the pandemic) were due to the pandemic or reflected previously existing levels of anxiety and insomnia among nurses.

-The results relate to the time period when the study was conducted under the specific conditions, so caution should be exercised when interpreting or generalizing the results.

-Data collection was performed through self-report questionnaires rather than clinical interviews or objective data measuring insomnia to confirm subjective reports. Also, to minimize bias that may be due to self-report questionnaires, confidentiality and anonymity were ensured to the maximum extent possible by the appropriate method of distribution and collection of the questionnaires.

-The study's limitations, including its cross-sectional nature, reliance on self-report questionnaires and lack of clinical interviews or objective data to confirm subjective reports, should be considered when interpreting or generalizing the results.

-While the study highlights the protective role of family support against negative psychological conditions, including insomnia and anxiety, it would be useful to investigate other sources of social support, such as colleagues or friends, in future studies.

-The study's findings could be used to inform the development of support systems to manage anxiety and insomnia in nurses, particularly for female nurses, who seem to be more vulnerable than their male colleagues and constitute the majority of nursing staff.

## 7. Conclusions

In conclusion, two years after the onset of the pandemic crisis, nurses were found to show high levels of anxiety, worsening insomnia and a concomitant decrease in family support compared to the first year of the pandemic. Insomnia seems to be significantly dependent on state anxiety, with a significant indirect effect of trait anxiety, while family support seems to influence state anxiety. State anxiety, trait anxiety and insomnia seem to show high positive correlations with each other, while all three parameters showed high negative correlations with family support, highlighting its protective role. Because the COVID-19 pandemic is still ongoing, with unknown long-term psychological effects on nurses, programs should be developed and implemented to manage anxiety and insomnia in nurses.

Particularly for female nurses, who seem to be more vulnerable than their male colleagues and who also constitute the majority of nursing staff, immediate support measures should be taken to protect their physical and mental health.

The findings of our study can be used as reference data for the development of an effective support system to reduce stress and improve family support and sleep health of nurses.
